# Handling linkage disequilibrium in qualitative trait linkage analysis using dense SNPs: a two-step strategy

**DOI:** 10.1186/1471-2156-10-44

**Published:** 2009-08-10

**Authors:** Kelly Cho, Josée Dupuis

**Affiliations:** 1 Department of Biostatistics, Boston University School of Public Health, Boston, MA, USA; 2 Current address: Departments of Genetics and Biostatistics, Yale University Schools of Medicine and Public Health, PO BOX 208034, 300 George Street Suite 523, New Haven, CT 06520-8034, USA

## Abstract

**Background:**

In affected sibling pair linkage analysis, the presence of linkage disequilibrium (LD) has been shown to lead to overestimation of the number of alleles shared identity-by-descent (IBD) among sibling pairs when parents are ungenotyped. This inflation results in spurious evidence for linkage even when the markers and the disease locus are not linked. In our study, we first theoretically evaluate how inflation in IBD probabilities leads to overestimation of a nonparametric linkage (NPL) statistic under the assumption of linkage equilibrium. Next, we propose a two-step processing strategy in order to systematically evaluate approaches to handle LD. Based on the observed inflation of expected logarithm of the odds ratio (LOD) from our theoretical exploration, we implemented our proposed two-step processing strategy. Step 1 involves three techniques to filter a dense set of markers. In step 2, we use the selected subset of markers from step 1 and apply four different methods of handling LD among dense markers: 1) marker thinning (MT); 2) recursive elimination; 3) SNPLINK; and 4) LD modeling approach in MERLIN. We evaluate relative performance of each method through simulation.

**Results:**

We observed LOD score inflation only when the parents were ungenotyped. For a given number of markers, all approaches evaluated for each type of LD threshold performed similarly; however, RE approach was the only one that eliminated the LOD score bias. Our simulation results indicate a reduction of approximately 75% to complete elimination of the LOD score inflation while maintaining the information content (IC) when setting a tolerable squared correlation coefficient LD threshold (r^2^) above 0.3 for or 2 SNPs per cM using MT.

**Conclusion:**

We have established a theoretical basis of how inflated IBD information among dense markers overestimates a NPL statistic. The two-step processing strategy serves as a useful framework to systematically evaluate relative performance of different methods to handle LD.

## Background

With rapid development of high throughput genotyping technologies, researchers have begun genome-wide searches for genes associated with complex diseases using dense single nucleotide polymorphisms (SNPs). With increased marker density, there is an increase in the likelihood that SNPs will be in linkage disequilibrium (LD), where some combinations of alleles or genetic markers inherently occur more frequently than would be expected at random. However, most multipoint linkage methods assume linkage equilibrium (LE). Among dense SNPs, the assumption of LE in linkage analysis may lead to incorrect pedigree haplotype inference [1,2] with unavailable parent genotypes. Moreover, several studies [3-6] have shown that LE assumption among tightly linked markers induces false-positive evidence for linkage in qualitative trait linkage analysis with ungenotyped parents This bias is influenced by SNPs in LD, which causes apparent oversharing of multipoint identity by descent (IBD) under the assumption of LE. Thus appropriate LD modeling or adjusting for markers in LD is necessary to avoid the upward bias in linkage when parental genotypes are not available.

In our study, we evaluate methods to handle LD in order to make recommendations on the best methods and tolerable LD thresholds to use in qualitative linkage analysis. We first present a theoretical evaluation of IBD sharing estimation under the incorrect assumption of LE and of the resulting inflation in linkage evidence in affected sib pair (ASP) analysis using a nonparametric linkage (NPL) statistic. We then describe different methods to handle LD achieved through a two-step processing strategy. The first step is an initial filtering where we applied three techniques: 1) removal of uninformative markers; 2) removal of redundant markers; 3) a combination of 1 and 2. In the second step, we examine and compare the following four approaches of handling LD in ASP linkage analysis: 1) a marker thinning (MT) algorithm; 2) a recursive elimination (RE) algorithm to select the most informative markers in LE; 3) SNPLINK [7]; and 4) MERLIN-LD [8]. Then we perform a simulation study using a dense set of markers similar in allele frequency and density to the Affymetrix GeneChip^® ^500 K set of SNPs using information from the subset of SNPs included in The HapMap Project to evaluate the relative performance of these various approaches in handling LD.

## Methods

### ASP Linkage Analysis

There are several methods available to perform multipoint linkage analysis using ASP, including maximum likelihood-based methods and the NPL analysis. The maximum likelihood-based approach is a powerful method but is sensitive to misspecification of the true mode of inheritance [9,10]. On the other hand, the NPL statistic relies on a measure (or score) of sharing among affected members. Whittemore and Halpern [11] proposed two scoring functions, *S*_*pairs *_and *S*_*all*_, which were implemented by Kruglyak et al[12]. *S*_*pairs *_is based on the number of distinct alleles shared IBD by affected members. If there is more than one affected sibling pair in a family, *S*_*all *_assigns increased score values based on the joint patterns of genetic transmission in all affected individuals in a family. For ASP, both scoring functions are equivalent; thus we consider *S*_*pairs *_in our investigation of the effect of LD on NPL statistics.

To be more explicit, we denote α_0_, α_1 _and α_2 _as the probabilities of sharing 0, 1 or 2 alleles IBD. When the markers are in LE and under H_0_, both α_2 _and α_0 _are equal to 1/4; thus . However, if LD is present but is not taken into consideration, the presence of LD inflates the estimate of α_2 _() to be great than 1/4, and the estimate of α_0 _() will generally be < 1/4; thus such overestimation of the proportion of alleles shared IBD causes the inflation of NPL LOD scores in the ASP linkage analysis. In addition, this inflation in the NPL LOD scores increases as the sample size (*n*) increases. When accounting for LD between markers, there is no inflation and *E(LOD) *= 0.11; note that the expected value under H_0 _is greater than 0 because the NPL LOD score cannot be negative. However when we ignore the presence of LD in the linkage analysis,  decreases, and at the same time, inflates, resulting in overall inflated *E(LOD)*. Therefore, if LD is not properly accounted for in the linkage analysis, it results in LOD score bias and hence the excess type-I error. We next propose and implement a strategy to minimize the excess type-I error in ASP analysis with markers in LD.

### Two-Step Strategy

#### Step 1

Step 1 allows us to study the degree of reduction in LOD score bias and IC by systematically filtering markers. In practice, working with a more manageable number of markers increases computational efficiency when performing multipoint linkage analysis. In an attempt to filter the dense set of markers, we apply three techniques: 1) removal of uninformative markers; 2) removal of redundant markers; and 3) a combination of 1 and 2. These techniques have been widely used in practice; however all three have not been systematically and formally evaluated together.

The first technique removes SNPs which are not very informative for linkage analysis, as measured by the minor allele frequency, MAF. A subset of SNPs with MAF above a pre-defined threshold is used in linkage analysis. We apply three MAF thresholds: 5%, 10% and 20% to the dense set of markers. The second technique removes SNPs with redundant information, as defined using r^2 ^≥ 0.95, where r^2 ^is a pairwise LD measure between two markers. A perfect correlation (r^2 ^= 1) indicates that a single SNP from the pair is sufficient to capture all of the information provided by the SNP pair. We omit one of the SNPs in each pair using the r^2 ^threshold. The third technique is a combination of the first two techniques. We apply 5% MAF threshold in conjunction with removal of redundant SNPs.

The three techniques together produce a total of 5 subsets of markers to compare: 3 subsets removing SNPs with low MAF (5, 10 or 20%); 1 subset without redundant SNPs; and 1 subset removing SNPs with 5% MAF and without redundant SNPs. We perform linkage analysis using these 5 subsets in families with 2, 3 or 4 affected siblings with and without genotyped parents. For each marker subset, we evaluate the LOD scores and the IC to measure relative performance in terms of reducing the LOD score bias and loss of IC compared to using the full set of markers.

#### Step 2

In step 2, we use the selected baseline subset of SNPs from step 1 with a reduced number of markers but with close to full IC to evaluate relative performance of four different methods to handle LD using varying LD cut points in the ASP linkage analysis. The four approaches are: 1) MT algorithm, 2) RE algorithm, 3) SNPLINK and 4) MERLIN-LD. Two of the four approaches, SNPLINK and MERLIN-LD, are currently available approaches to handle LD and have been used in other studies. The MT algorithm has been suggested as a simple way to deal with LD in datasets [13-15]. We propose the RE algorithm to eliminate markers in LD while retaining the most informative markers. Descriptions of these four approaches are summarized below.

#### Marker Thinning (MT) Algorithm

MT algorithm utilizes various pre-specified marker densities to select SNPs over the entire chromosome of interest. This approach thins out dense mapping by decreasing the SNP density to be analyzed. Depending on the density level and the chromosome length, one SNP is chosen at each interval creating a smaller subset of SNPs. We apply four different density thresholds: 1, 2, 4 and 8 SNPs per 1 cM region.

#### Recursive Elimination (RE) Algorithm

We propose the RE algorithm to combine the informativeness of each marker with a measure of LD (either D' or r^2^) to select a subset of markers to perform linkage analysis. Markers with a higher MAF are more informative in linkage analysis because individuals are more likely to carry heterozygous genotypes; segregation from homozygous individuals cannot be determined unambiguously. Using a user-specified LD threshold, this approach first takes a pair of SNPs above the given LD threshold and removes the less informative marker from the pair. This procedure is repeated iteratively until no pairwise LD measures exceed the threshold. The resulting subset of SNPs contains a reduced number of SNPs without pairwise LD above a given threshold. The resulting subset may have reduced information content despite the attempt to keep the most informative markers from each pair of marker in LD.

#### SNPLINK

SNPLINK is a Perl script created to undertake automated genetic analyses and to address the issue of LD [7]. SNPLINK takes a user-defined LD threshold by D', r^2 ^or the combination of the two measures. Then markers are first grouped into sets, where each consecutive marker pair in the set is found to be in LD above a specified threshold. LD is handled in a straight forward fashion by selecting the middle SNP from each set of markers in LD. This results in a new LD-reduced set of SNPs ready for further analysis. However, removing SNPs using SNPLINK may reduce information content. In addition, SNPLINK takes a conservative approach to simplify the estimation of linkage phase of the SNPs, which is not directly observed. It does this by ignoring the relationships among family which may jeopardize the accuracy of the estimation.

#### MERLIN-LD

MERLIN-LD refers to an efficient approach of modeling LD that has been implemented in the MERLIN software package [16]. This approach groups tightly linked markers into clusters. The current implementation of the MERLIN software package is capable of taking either the physical distance or r^2 ^as the measure of LD to form clusters. MERLIN-LD creates a cluster by joining pairs of markers for which the LD measure exceeds a pre-specified threshold with all intervening markers or markers within a certain distance of each other if the distance option is used. Some limitations of MERLIN-LD include that the current implementation does not allow the use of D' as the LD threshold, and that the approach assumes no recombination within clusters and no LD between clusters, which may not hold in practice.

### Simulation

Our simulation is based on the LD structure observed in the HapMap database and a highly dense SNP panel from the 500 K Affymetrix array. We obtained the HapMap phased haplotypes for all 6,012 available HapMap SNPs from the 500 K SNP Affymetrix array on chromosome 21. We selected chromosome 21 in our simulation study because of a previous report of LOD score inflation due to LD in this region [17].

Using the set of 120 haplotypes from the HapMap CEPH 30 trio family data, we first introduce recombination according to the Haldane mapping function that mimics approximately eight generations of crossovers and generate a new pool of haplotypes. The newly generated haplotypes are randomly assigned to all parents. Crossovers are generated along the parental chromosomes with a rate of 1 crossover per Morgan [18]. to create two gametes for each parent: the first gamete comprises half of the parental alleles, and the second gamete includes the other half. Under the null hypothesis of no linkage, each offspring then inherits one of the two gametes at random from each parent. There are a total of 9 scenarios in our study design: families of 2 (n = 500), 3 (n = 300) or 4 (n = 100) affected siblings with additional 0, 1 or 2 unaffected siblings.

In our investigation of the effects of LD, we use varying thresholds or cut points of two widely used measures of LD (D' and r^2^), where applicable, to show a gradual change or trend of LOD score inflation along the ranges of LD measures. The following sets of LD thresholds by D' and r^2 ^are applied in handling LD: 0.1, 0.3, 0.5, and 0.7.

## Results

We evaluated different methods to handle LD in the framework of our proposed two-step processing strategy. In step 1, we performed NPL analysis over 1,000 replicates of the simulated data at different LD thresholds for families with 2, 3 or 4 affected siblings and zero or two ungenotyped parents to determine which subset of SNPs retained full IC while reducing the LOD score bias. In step 2, we performed NPL analysis over 1,000 replicates of the simulated data at different LD thresholds using the subset of SNPs obtained from step 1 for all 9 study designs, with zero or two ungenotyped parents. We evaluated different approaches at varying degrees of LD (D' and r^2^) in terms of reducing the LOD score bias. In general, with complete data where both parents are genotyped, there was no LOD score inflation in all study designs. Thus our discussions to follow mainly focus on those results where both parents are ungenotyped.

### Step 1

Table 1 summarizes average maximum LOD score (MLS) and average IC obtained using the three techniques as they compared to the unadjusted subset of SNPs. The IC values obtained at unadjusted subset of SNPs were 0.72, 0.80 and 0.84 for the 2, 3 or 4 sibling data, respectively.

**Table 1:** Step 1: Summary descriptive statistics of the average maximum NPL LOD scores and the average IC with ungenotyped parents.

		2 affected sibs		3 affected sibs		4 affected sibs	
	Ave # SNPs	MLS (SD)	IC	MLS (SD)	IC	MLS (SD)	IC
Unadjusted	6012	13.27 (2.77)	0.72	14.41 (3.64)	0.80	7.86 (2.20)	0.84
MAF ≥ 0.05	5387	13.49 (2.66)	0.72	14.77 (3.64)	0.80	8.06 (2.23)	0.84
MAF ≥ 0.10	4616	13.87 (2.64)	0.72	15.00 (3.61)	0.80	8.18 (2.25)	0.84
MAF ≥ 0.20	3203	15.01 (2.69)	0.72	15.47 (3.61)	0.79	7.90 (2.42)	0.84
r^2 ^≥ 0.95	4596	5.47 (1.79)	0.72	4.93 (2.06)	0.80	2.96 (1.55)	0.85
MAF ≥ 0.05 & r^2 ^≥ 0.95	3713	9.62 (2.75)	0.72	14.01 (3.43)	0.80	5.02 (1.95)	0.85

Removing uninformative SNPs reduced the number of markers while maintaining the level of IC; however, the LOD score inflation remained unchanged compared to the baseline LOD score inflation. Removing redundant SNPs resulted in no loss of IC and showed moderate reduction of the LOD score bias and outperformed the combination technique; however there was still a large number of markers selected. When the two techniques were combined, 38% fewer SNPs than the baseline subset of SNPs were selected while maintaining IC. In addition, this combination technique showed moderate reduction in LOD score bias. In general, for all marker subsets, the average MLS remained above the null expectation, pointing out the need for further adjustment/SNP selection to reduce the bias introduced by ignoring LD in dense marker sets. We carried forward the marker subset created by the combination approach to be evaluated in step 2 because we achieved the same information content while reducing bias. Setting the results from the combination approach in Step 1 as the baseline for Step 2 allowed us to evaluate the different approaches and measure the level of bias each approach could reduce by starting from a moderate baseline of bias.

### Step 2

In step 2, we examined four different approaches to handle LD among dense SNPs using the selected subset of 3,713 SNPs in step 1 as the baseline marker subset. We compared the resulting average MLS from these different approaches to the baseline and considered them as showing reduction of bias where we noted the observed average MLS at least 10% below the baseline. Table 2 shows the average MLS with ungenotyped parents, where LOD score bias was observed. As a note, with the addition of one or two unaffected siblings, we observed lower LOD score bias as shown in Table 1. For example, we observed 9.62 (SD = 2.75), 8.66 (SD = 2.49) and 4.41 (SD = 1.80) average MLS for families of 2 affected sibs and zero, one or two unaffected sibs, respectively, with ungenotyped parents. Similar trends were observed for other study designs with 3 or 4 affected siblings. In term of IC, we observed increased average IC as the size of a sibship in a family increased.

**Table 2:** Summary of average maximum LOD scores and average IC using the baseline marker subset from step 1 for the 9 study designs with ungenotyped parents.

Number of Sibs	MLS (SD)	Average IC
2 Affected	9.62 (2.75)	0.72
3 Affected	14.01 (3.43)	0.80
4 Affected	5.02 (1.95)	0.85
2 Affected + 1 Unaffected	8.66 (2.49)	0.80
3 Affected + 1 Unaffected	5.41 (2.12)	0.85
4 Affected + 1 Unaffected	3.17 (1.55)	0.88
2 Affected + 2 Unaffected	4.41 (1.80)	0.85
3 Affected + 2 Unaffected	3.18 (1.57)	0.88
4 Affected + 2 Unaffected	1.89 (1.29)	0.90

In Table 3 and 4, we summarize the average MLS using the four methods in handling LD at varying LD cut points using families with 2, 3 or 4 affected siblings and ungenotyped parents. Table 3 shows results obtained using D' thresholds, and Table 4 shows results obtained using r^2 ^thresholds for families with 2, 3 or 4 affected siblings with ungenotyped parents. In addition, similar patterns across the LD cut points were observed with further reduction of the LOD score bias for families with 1 or 2 additional siblings who were not affected (data not shown). As the number of unaffected siblings increased in a family the inflation of LOD scores diminished further and in some cases were eliminated completely.

**Table 3:** Step 2 using D' LD threshold and MT: Summary descriptive statistics of average maximum NPL LOD scores and average IC for families with 2, 3 or 4 affected sibling and ungenotyped* parents.

			2 affected sibs		3 affected sibs		4 affected sibs	
Method	LD threshold	Ave # SNPs	MLS (SD)	IC	MLS (SD)	IC	MLS (SD)	IC
Unadjusted		3713	9.62 (2.75)	0.72	14.01 (3.43)	0.80	5.02 (1.95)	0.85
MT	8snp1cM	480	1.20 (0.96)	0.72	1.34 (1.01)	0.80	0.36 (0.47)	0.85
MT	4snp1cM	259	0.65 (0.67)	0.71	0.60 (0.63)	0.80	0.22 (0.36)	0.85
MT	2snp1cM	135	0.66 (0.68)	0.67	0.76 (0.74)	0.79	0.26 (0.38)	0.84
MT	1snp1cM	68	0.76 (0.73)	0.61	1.05 (0.88)	0.76	0.34 (0.47)	0.83
RE	0.7	409	0.44 (0.53)	0.73	0.54 (0.61)	0.80	0.2 (0.33)	0.85
RE	0.5	309	0.37 (0.50)	0.72	0.45 (0.55)	0.80	0.18 (0.34)	0.85
RE	0.3	200	0.43 (0.54)	0.7	0.58 (0.60)	0.80	0.23 (0.39)	0.85
RE	0.1	62	0.73 (0.73)	0.61	1.18 (0.96)	0.76	0.44 (0.57)	0.83
SNPLINK	0.7	531	0.65 (0.78)	0.73	0.66 (0.79)	0.80	0.3 (0.46)	0.85
SNPLINK	0.5	401	0.75 (0.81)	0.72	0.73 (0.83)	0.80	0.31 (0.46)	0.85
SNPLINK	0.3	287	0.85 (0.88)	0.71	0.81 (0.84)	0.80	0.33 (0.47)	0.85
SNPLINK	0.1	120	0.65 (0.74)	0.64	0.70 (0.81)	0.77	0.31 (0.46)	0.83

*With complete data where both parents are genotyped, the unadjusted average MLS for 2, 3 or 4 affected sibs are 0.58, 0.5 and 0.47.

**Table 4:** Step 2 using r^2 ^LD threshold: Summary descriptive statistics of average maximum NPL LOD scores and average IC for families with 2, 3 or 4 affected sibling and ungenotyped* parents.

			2 affected sibs		3 affected sibs		4 affected sibs	
Method	LD threshold	Ave # SNPs	MLS (SD)	IC	MLS (SD)	IC	MLS (SD)	IC
Unadjusted		3713	9.62 (2.75)	0.72	14.01 (3.43)	0.80	5.02 (1.95)	0.85
RE	0.7	1562	1.39 (0.98)	0.73	2.00 (1.28)	0.80	0.73 (0.70)	0.85
RE	0.5	1240	1.21 (0.90)	0.73	1.67 (1.17)	0.81	0.59 (0.63)	0.85
RE	0.3	892	0.45 (0.53)	0.73	0.66 (0.68)	0.81	0.26 (0.39)	0.85
RE	0.1	435	0.32 (0.44)	0.72	0.40 (0.49)	0.80	0.18 (0.34)	0.85
MERLINLD	0.7	562	1.35 (0.97)	0.73	1.83 (1.22)	0.80	0.62 (0.61)	0.85
MERLINLD	0.5	575	1.12 (0.96)	0.73	1.53 (1.19)	0.80	0.53 (0.59)	0.85
MERLINLD	0.3	542	0.55 (0.61)	0.74	0.77 (0.77)	0.80	0.32 (0.43)	0.85
MERLINLD	0.1	423	0.51 (0.58)	0.74	0.69 (0.70)	0.81	0.29 (0.41)	0.85
SNPLINK	0.7	2596	3.94 (1.85)	0.73	5.10 (2.11)	0.80	2.06 (1.23)	0.85
SNPLINK	0.5	2351	3.63 (1.81)	0.73	4.88 (2.09)	0.80	1.97 (1.20)	0.85
SNPLINK	0.3	2057	3.04 (1.73)	0.73	4.10 (1.95)	0.80	1.56 (1.07)	0.85
SNPLINK	0.1	1519	2.69 (1.64)	0.73	3.57 (1.87)	0.80	1.28 (0.98)	0.85

*With complete data where both parents are genotyped, the unadjusted average MLS for 2, 3 or 4 affected sibs are 0.58, 0.5 and 0.47

#### Marker Thinning (MT) Algorithm

For the four MT cut points with 8, 4, 2 or 1 SNPs per 1 cM region, we observed the average MLS of 1.20 (SD = 0.96), 0.65 (SD = 0.67), 0.66 (SD = 0.68) and 0.76 (SD = 0.73), respectively (Table 3). The corresponding average IC values were 0.72, 0.71, 0.67 and 0.61, respectively. Compared to the baseline LOD score of 9.62 (SD = 2.75), all four subsets of markers resulted in greatly reduced LOD score bias. At 8 and 4 SNPs per cM cut points, there was negligible loss of information; however, at the other two lower cut points, there was some loss of information compared to the baseline IC of 0.72. Nevertheless, none of these sets of markers completely eliminated the LOD score bias, as compared to the average MLS values observed with complete data where both parents are genotyped. Similar trends were observed for families with 3 or 4 siblings.

#### Recursive Elimination (RE) Algorithm

Using D' cut points, we observed a substantial reduction of the LOD score bias as compared to the baseline LOD score of 9.62 (SD = 2.75) shown in Table 3. Compared to the observed average MLS with complete data, using RE approach eliminated the bias at D' > 0.3, but we did not observe the same pattern at the lowest D' cut point. Some loss of information was observed at D' 0.3 (IC = 0.70) and 0.1 (IC = 0.61) cut points compared to the baseline. With r^2 ^thresholds applied to the data with 2 affected siblings, we also observed reduction of the LOD score bias for the four r^2 ^cut points, respectively (Table 4). When compared to the complete data, the LOD score bias was no longer observed at the two lowest r^2 ^cut points. In addition, the IC values did not change from the baseline IC of 0.72. In general for both LD measures, similar trends were observed for families with 3 or 4 affected siblings.

#### SNPLINK

Compared to the baseline LOD score of 9.62, SNPLINK adjusted marker subsets reduced the LOD score bias when using D' thresholds (Table 3); however none of the cut points completely eliminated the bias. There was some information loss only at the lowest cut point (IC = 0.64) compared to the baseline IC of 0.72. In contrast, using r^2 ^thresholds, we observed only moderate reduction of the LOD score bias (Table 4), and there was no loss of information at any level of threshold. Relatively similar patterns of reduction were observed in families with 3 or 4 siblings across all cut points for both LD measures.

#### MERLIN-LD

We applied the clustering method implemented in MERLIN, MERLIN-LD, using four r^2 ^cut points. There were 562, 575, 542 and 423 clusters formed at 0.7, 0.5, 0.3 and 0.1 r^2 ^cut points, respectively (Table 4). Across all thresholds, the IC remained unchanged from the baseline IC. For families with 2 affected siblings and ungenotyped parents, we observed a substantial reduction of LOD score bias as compared to the baseline LOD score of 9.62 (Table 4). At the two lowest cut points, we observed elimination of the LOD score bias as they compared to the average MLS with complete data.

## Discussion

Overestimation of IBD sharing in ASP analysis causes upward bias in qualitative linkage analysis. This overestimation of IBD sharing is caused by ignoring LD among dense markers with ungenotyped parents. Most commonly used multipoint linkage methods assume LE among markers and ignore the possibility that LD could be present. The underlying assumption of LE is violated when using dense mapping in linkage analysis. As a result, we observe spurious evidence of linkage even when the markers are unlinked to the disease locus. This paper focuses only on inflation of nonparametric linkage statistics under the null hypothesis. For the approaches which eliminated the bias under the null, we did not investigate which approach was most powerful. However, the reduction in IC may give some indication as the effect on power, with method preserving IC being more desirable.

In our study, we theoretically explored how inflated IBD sharing estimates among sibling pairs affect the NPL statistic under the null hypothesis of no linkage. When properly accounting for LD, the estimated probabilities of observing 0 and 2 alleles IBD, denoted as  and , are each asymptotically equal to 1/4. However under the assumption of LE,  is less than 1/4, and  is greater than 1/4. We showed how these changes in  and  have direct affect on the NPL statistic, inflating the LOD score under the assumption of LE. Using the two marker example with complete LD, we illustrated inflated LOD scores by ignoring LD.

Next we proposed a two-step processing strategy for reducing the bias observed by ignoring LD among dense SNPs, while preserving the IC. We then evaluated our two-step processing strategy using a combination of the dense Affymetrix 500 K SNPs and the SNPs available in the HapMap database for chromosome 21. There were 6,012 SNPs in this set. We performed a simulation study using ASP data with 2 (n = 500), 3 (n = 200) or 4 (n = 100) affected sibs. We analyzed the data with and without genotyped parents. Additional study designs were examined by adding 1 or 2 unaffected sibs to the first three scenarios. Through a simulation study, we investigated the relative performance of our two-step processing strategy to handle LD among dense markers against the baseline LOD score inflation and the level of IC.

The two-step strategy has enabled us to evaluate methods to handle LD in terms of reducing the upward bias. Step 1 filters the marker map while keeping the IC which reduced a burden of computation intensity and time. Step 2 systematically evaluates different approaches to handle LD. In general, we reduced approximately 75% to complete elimination of the LOD score inflation while maintaining the IC when setting a tolerable LD threshold above r^2 ^0.3 or 2 SNPs per cM using the MT algorithm. In addition, fewer markers were selected using D' compared to using r^2^, and we observed more reduction of the LOD score bias using r^2 ^compared to using D' in our data for RE and SNPLINK approaches. For a given number of markers, RE, SNPLINK and MT methods performed at a similar level using D' threshold; however, RE approach was the only one that eliminated the LOD score bias. As a note, we found that the estimates become unstable when the number of makers analyzed falls below a certain level. This is apparent using D' threshold, where we observed that average MLS for both MT (1snp1cM) and RE (0.1) thresholds show elevation compared to the higher thresholds (Table 3). In both instances, the number of markers analyzed is below 70, and these estimates have large variances. For a given number of markers using r^2 ^thresholds, RE and SNPLINK performed similarly in reducing the LOD score bias, while RE completely eliminated the bias at the two lowest cut points. MERLIN-LD performed as well as RE (r^2^) in reducing the LOD score bias. As the cut points decreased, we observed increased reduction of bias; nevertheless, at lower cut points, some loss of information was observed.

Through our investigation of the effect of LD in qualitative trait linkage analysis, we learned that inflation of LOD score depends on the following factors: ungenotyped parents, sample size, study design and the structure of the underlying LD in the dataset. In addition, the inflation increases as more markers in LD are considered in multipoint linkage analysis [3,5]. In terms of the number of SNPs in our simulation, fewer markers were selected when using the D' LD threshold compared to the r^2 ^cut points. Moreover, Boyles et al [3] concluded that r^2^was superior in terms of predicting inflation compared to D' in their study. Different samples appear to have slight variations in thresholds of LD measures and in the number of tolerable SNPs per cM that eliminate the inflation of LOD score [5,6]. Therefore, choosing an appropriate measure of LD in handling LD, in general, depends on the underlying LD structure which is unique to each dataset. Methods that are robust to the various underlying LD structures are of great interest for further investigation.

## Conclusion

Overall in our study, we have established the theoretical basis for the effect of LD between markers on linkage statistics for qualitative traits. We have proposed and implemented a two-step processing strategy to systematically minimize the impact of LD in linkage analysis and to evaluate different methods in handling LD. Finally, we have made recommendations on appropriate methods and LD thresholds that minimize the impact of LD among dense SNPs. In summary, for a given number of markers, all approaches evaluated for each type of LD threshold performed similarly; however, RE approach was the only one that eliminated the LOD score bias. In general, approximately 75% to complete elimination of the LOD score inflation can be reached while maintaining the IC when setting a tolerable LD threshold above r^2 ^0.3 or 2 SNPs per cM using the MT algorithm. Our results encourage further research in development of methods that combine the flexibility in handling LD with ease of application.

With rapidly advancing genotyping techniques and decreasing cost, the field of genetic mapping is moving towards genotyping even denser markers than currently used 500 K set of markers. With increasing volume of available genotypes, there is also an increasing demand and interest in dealing with marker selection and linkage disequilibrium among dense markers in linkage analysis. The need and efforts in developing and investigating new algorithms and approaches to appropriately and manageably handle all markers on the finest scaled mapping will continue to grow in the field of genetic mapping and analyses.

## Authors' contributions

KC participated in the design of the study, interpretation of results, performed the simulation studies and drafted the paper. JD contributed substantially to the conception and design of the study, to the interpretation of results and revised the manuscript. Both authors read and approved the final manuscript.
